# Regional differences in the link between water exchange rate across the blood–brain barrier and cognitive performance in normal aging

**DOI:** 10.1007/s11357-023-00930-2

**Published:** 2023-09-15

**Authors:** Valentinos Zachariou, Colleen Pappas, Christopher E. Bauer, Xingfeng Shao, Peiying Liu, Hanzhang Lu, Danny J. J. Wang, Brian T. Gold

**Affiliations:** 1 Department of Neuroscience, College of Medicine, University of Kentucky, Lexington, KY USA; 2 Laboratory of FMRI Technology (LOFT), Mark & Mary Stevens Neuroimaging and Informatics Institute, Keck School of Medicine, University of Southern California, Los Angeles, CA USA; 3 Department of Diagnostic Radiology & Nuclear Medicine, University of Maryland School of Medicine, Baltimore, MD USA; 4 Department of Radiology, Johns Hopkins University School of Medicine, Baltimore, MD USA; 5 Sanders-Brown Center On Aging, University of Kentucky, Lexington, KY USA; 6 Magnetic Resonance Imaging and Spectroscopy Center, University of Kentucky, Lexington, KY USA

**Keywords:** BBB water exchange rate, Normal aging, Cognitive performance, Biological mechanisms

## Abstract

The blood–brain barrier (BBB) undergoes functional changes with aging which may contribute to cognitive decline. A novel, diffusion prepared arterial spin labeling-based MRI technique can measure the rate of water exchange across the BBB (*k*_w_) and may thus be sensitive to age-related alterations in water exchange at the BBB. However, studies investigating relationships between *k*_w_ and cognition have reported different directions of association. Here, we begin to investigate the direction of associations between *k*_w_ and cognition in different brain regions, and their possible underpinnings, by evaluating links between *k*_w_, cognitive performance, and MRI markers of cerebrovascular dysfunction and/or damage. Forty-seven healthy older adults (age range 61–84) underwent neuroimaging to obtain whole-brain measures of *k*_w_, cerebrovascular reactivity (CVR), and white matter hyperintensity (WMH) volumes. Additionally, participants completed uniform data set (Version 3) neuropsychological tests of executive function (EF) and episodic memory (MEM). Voxel-wise linear regressions were conducted to test associations between *k*_w_ and cognitive performance, CVR, and WMH volumes. We found that *k*_w_ in the frontoparietal brain regions was positively associated with cognitive performance but not with CVR or WMH volumes. Conversely, *k*_w_ in the basal ganglia was negatively associated with cognitive performance and CVR and positively associated with regional, periventricular WMH volume. These regionally dependent associations may relate to different physiological underpinnings in the relationships between *k*_w_ and cognition in neocortical versus subcortical brain regions in older adults.

## Introduction

Normal aging is associated with alterations in cerebrovascular function that negatively affect cognitive performance [1–4]. An increasing number of studies indicate that disruption of the blood–brain barrier (BBB) may represent an early contributor to age-related vascular cognitive declines [5–10]. The BBB is a highly regulated vascular interface between the blood and central nervous system. It is made up of the endothelial cells that limit permeability and is sheathed by perivascular mural cells, pericytes, and astrocytic endfeet [5, 11, 12]. The endothelial cells of the BBB have tight junctions which serve to regulate the flow of ions and molecules between the blood and brain and prevent toxins and pathogens from entering the brain, supporting proper neuronal functioning [13, 14].

BBB disruption can be assessed in vivo using imaging techniques such as dynamic contrast‐enhanced magnetic resonance imaging (DCE MRI [5]). DCE MRI can track paracellular leakage of gadolinium (Gd)-based contrast agents (GBCAs) as these pass between the blood and brain (*k*_trans_), revealing important information about both advanced and more subtle tight junction disruption [5, 15, 16], as well as increases in transcellular bulk flow transcytosis across the BBB [17]. Increased *k*_trans_ is associated with cognitive dysfunction in mild cognitive impairment, Alzheimer’s disease, and vascular cognitive impairment [7, 18, 19]. However, GBCAs have relatively large molecular weights (Gd‐DTPA 550 Da) which may necessitate significant structural damage to the BBB before extravasation occurs [5, 20, 21].

Recently, a non-invasive MRI method has been proposed to measure subtler BBB alterations in the transport of water across the BBB using diffusion prepared arterial spin labeling (DP-ASL) MRI [21–23]. Unlike DCE-MRI, the DP-ASL technique does not measure alterations in the permeability of the BBB. Instead, it can estimate water exchange rate across the BBB (expressed as *k*_w_) which indicates the rate of water molecule exchange between capillaries and brain tissue (i.e., reciprocal of water exchange time) [21]. The majority of water molecules traverse the BBB via water-specific transport proteins known as aquaporins, with aquaporin-4 (AQP4) being the most prevalent aquaporin channel in the brain [24]. Characterized by a diameter comparable to that of a single water molecule, AQP4 only allows for diffusion of one molecule at a time [24, 25]. This regulated water transfer across the BBB has physiological significance in safeguarding the brain against edema and swelling [26, 27]. DP-ASL has been validated in relation to the exchange of ASL tagged water with tissue water in the rat brain, using a hypercapnic challenge [28, 29]. For instance, in the rat brain BBB *k*_w_ was shown to increase as a function of mannitol-induced osmotic effects on water movement across the BBB [30].

This means that the MRI-based metric of DP-ASL *k*_w_ may be sensitive to decreases in cognitive performance associated with age-related alterations in water exchange across the BBB. To date, however, only a handful of studies have employed DP-ASL to investigate potential links between BBB *k*_w_ and cognition [21, 31–33]. In addition, the relationships reported between BBB *k*_w_ and cognitive performance have not been entirely consistent. In particular, while most studies have reported a positive relationship between BBB *k*_w_ and cognitive performance [31, 32], Shao et al. [21] reported negative associations between BBB *k*_w_ in whole brain, gray matter (GM), and white matter (WM) and cognitive performance in a cohort of older adults at risk of cerebral small vessel disease (cSVD).

Given the potential utility of BBB *k*_w_ as a non-invasive MRI metric of alterations in water exchange across the BBB, it is important to gain insight into the direction of associations between BBB *k*_w_ and cognitive function, as well as the underlying mechanisms involved. One possibility is that different physiological underpinnings may contribute to the direction of the *k*_w_–cognition associations. Previous research by our group and by other researchers hypothesized that the positive associations between BBB *k*_w_ and cognition may reflect optimal BBB-related protein clearance functions [31–33]. However, negative associations between BBB *k*_w_ and cognition appear incompatible with the optimal protein clearance function hypothesis. These negative associations were reported in a cohort of older adults at risk of cSVD [21]. Therefore, one possible explanation for these negative BBB *k*_w_–cognition associations could be age-related regional differences in cerebrovascular dysfunction and/or vascular related tissue damage.

In the current study, we investigate this possibility in a cohort of healthy older adults. Specifically, we first used a voxel-wise approach to identify associations between BBB *k*_w_ in all possible brain regions and composite measures of executive function (EF) and episodic memory (MEM). EF and MEM were selected because these cognitive domains are among the first to be affected in normal aging [34–36]. The voxel-wise analysis identified both positive and negative associations between BBB *k*_w_ and both EF and MEM, in different brain regions.

Second, to assess links between BBB *k*_w_ and cerebrovascular dysfunction and/or vascular-related tissue damage and how these links relate to cognitive performance, additional regression analyses were conducted between BBB *k*_w_ in all regions emerging from the voxel-wise analyses and 1) cerebrovascular reactivity (CVR), a validated marker of cerebrovascular compliance [37, 38] and 2) white matter hyperintensity (WMH) volumes, an MRI marker of WM damage associated with cSVD [39, 40].

## Materials and methods

### Participants

Forty-seven healthy older adults participated in this study. All participants provided informed consent under a protocol approved by the Institutional Review Board of the University of Kentucky. Participants were recruited from an existing longitudinal cohort at the Sanders–Brown Center on Aging [41] and the broader Lexington, KY, community. All participants were cognitively intact based on clinical consensus diagnosis and scores from the Uniform Data Set (UDS-3) used by US ADCs (procedure outlined in [42, 43]) or a score of 26 or higher on the Montreal Cognitive Assessment (MoCa [44]) for those participants recruited from the community.

Study exclusionary criteria were self-reported significant head injury (defined as loss of consciousness for more than five minutes), heart disease, neurological or psychiatric disorders, claustrophobia, pacemakers, the presence of metal fragments or any metal implants that are incompatible with MRI, diseases affecting the blood (anemia, kidney/heart disease, etc.), or significant brain abnormalities detected during imaging. Twenty-one of the participants self-reported hypertension, but indicated it was currently under control by prescription medication which they identified by name. Four participants not only indicated recent/active diabetes but also indicated it was currently under control by prescription medication which they identified by name. Detailed characteristics of the participant cohort are shown in Table 1. Thirty of the subjects in the current study also participated in [33].

**Table 1:** Group demographics and mean cognitive measures

*n*	47
M: F	21: 26
Hypertension medication (yes: no)	21: 26
Recent/active diabetes (yes: no)	4: 43
Age range (years)	61–84
Age (years)	70.6 $\pm$ 5.54^1^
Education (years)	16.5 $\pm$ 2.3^1^
MoCa^2^	27.78 $\pm$ 1.20^1^

^1^Mean $\pm$ standard deviation is shown for participants

^2^*MoCA* Montreal Cognitive Assessment

### Imaging protocol

Participants were scanned with a Siemens 3 T Prisma scanner (software version MR_VE11C), using a 64-channel head-coil, at the University of Kentucky Magnetic Resonance Imaging and Spectroscopy Center (MRISC). The following sequences were used: (1) a 3D multi-echo, T1-weighted, magnetization prepared rapid gradient echo sequence (ME-MPRAGE) used to define anatomical ROIs and for MNI152 template space normalization/warping [45]; (2) a 3D fluid-attenuated inversion recovery (FLAIR) sequence, used to evaluate white matter hyperintesity (WMH) volumes; (3) a blood oxygen level–dependent (BOLD), echo planar imaging (EPI), functional MRI (fMRI) scan for assessment of cerebrovascular reactivity (CVR); and (4) a 3D gradient-and-spin-echo (GRASE) diffusion-prepared pCASL (DP-pCASL) sequence for assessing the water exchange rate across the BBB *(k*_*w*_). Several other sequences were collected during the scanning session related to other scientific questions and are not discussed further here.

The multi-echo (ME)-MPRAGE sequence had four echoes (repetition time (TR) = 2530 ms; first echo time (TE1) = 1.69 ms echo time spacing (ΔTE) = 1.86 ms, flip angle (FA) = 7°) and covered the entire brain (176 slices, field of view = 256 mm, parallel imaging (GRAPPA), acceleration factor = 2, 1-mm^3^ isotropic voxels, scan duration = 5.53 min). A ME MPRAGE sequence was used because it optimizes gray/white matter contrast which improves the accuracy of FreeSurfer-based cortical/subcortical segmentations and subsequently the accuracy of delineated ROIs [46]. The 3D FLAIR sequence covered the entire brain and was acquired using the following parameters: 1-mm^3^ isotropic voxels, 256 × 256 × 176 acquisition matrix, TR = 5000 ms, TE = 388 ms, inversion time = 1800 ms, and scan duration = 6.45 min. The CVR scan also covered the entire brain and was acquired using a BOLD EPI sequence with the following parameters: voxel size of 3.0 × 3.0 × 3.7 mm^3^, acquisition matrix = 64 × 64 × 36, parallel imaging (GRAPPA) acceleration = 2, TR = 2000 ms, TE = 30 ms, FA = 71°, number of volumes = 216, and scan duration = 7.20 min.

Lastly, the DP-pCASL sequence was acquired with the following parameters: TR = 4 s, TE = 36.5 ms, FOV = 224 mm, matrix size = 64 × 64, 12 slices (10% oversampling), resolution = 3.5 × 3.5 × 8 mm^3^, label/control duration = 1500 ms, and centric ordering, and optimized timing of background suppression for grey matter (GM) and WM [47]. A two-stage approach was used to measure arterial transit time (ATT) and *k*_w_: fifteen repetitions were acquired during the flow encoding arterial spin tagging (FEAST) scan at post-labeling delay (PLD) = 900 ms and diffusion weighting (*b*-value) of 0 and 14 s/mm^2^ with a total acquisition time of 4 min for estimating ATT. The k_w_ metric was calculated from scans acquired at PLD = 1800 ms, when the labeled blood reaches the microvascular compartment, with *b* = 0 and 50 s/mm^2^, respectively. Twenty repetitions were acquired for each *b*-value of the *k*_w_ scan, and the total acquisition time was 6 min. The 12 slices of the DP-pCASL scan did not cover the entire brain. As such, before each DP-pCASL scan, the area of acquisition was manually positioned to include as much of the hippocampus as possible (98% average hippocampal coverage) without excluding any cortical brain regions. Inferior temporal lobe regions below the hippocampus were partially covered by the DP-pCASL scan (53% average inferior temporal lobe coverage).

### *BBB k*_*w*_* mapping*

BBB *k*_w_ maps were created using the procedure outlined in [21]. More specifically, DP-pCASL control/label images were first corrected for rigid head motion using SPM12 [48]. Residual motion artifacts resulting from temporal fluctuations in the control/label image acquisition were further reduced using principal component analysis [49], which can help restore the details of gyral structures. Following motion correction, *k*_w_ maps were calculated by a total–generalized–variation (TGV; [50]) regularized SPA model [23] using the tissue (or capillary) fraction of the ASL signal at the PLD of 1800 ms, incorporating ATT, T1 of arterial blood, and brain tissue as inputs for the algorithm [21]. Arterial blood T1 was assumed to be 1.66 s, which is commonly used for CBF quantification [51]. To highlight regional differences in BBB *k*_w_ across the participant cohort, the *k*_w_ maps were converted to *Z*-scores, representing the number of standard deviations that individual voxels deviated from the mean BBB *k*_w_ for each participant.

Following the creation of the BBB *k*_w_ maps, the four echoes of the T1-weighted structural images from the ME-MPRAGE scan were averaged into a root mean square (RMS) image and registered to the M0 image of the DP-pCASL scan. A semi-automatic, edge-based approach was then used for registration, implemented using the align_epi_anat function of AFNI [52]. In more detail, AFNIs 3dLocalstat function was first used to create normalized 2D gradient versions of the *k*_w_ and T1-weighted images, corresponding to the local spatial variance within each image. The local spatial variance was computed as the standard deviation of intensities within a local neighborhood of voxels, divided by their mean intensity. The optimal alignment was subsequently computed between the 2D gradient version of the T1-weighted/M0 images and the resulting transformation matrix was used to align the original T1/M0 images.

This edge-based alignment approach was selected because it yielded the best T1/M0 image registration among all the available methods provided by AFNI, for the majority of participants (inspected manually). For those participants in which registration was sub-optimal, the T1-weighted images were manually registered to the M0 images and corresponding transformation matrices created using the nudge tool in FSLeyes. Our semi-automated registration approach allowed for accurate registration of high-resolution T1-weighted images to the low-resolution images of the DP-pCASL scan (3.5 × 3.5 × 8 mm^3^).

Following registration, the BBB *k*_w_ registered T1-weighted images were non-linearly warped/normalized to MNI152 space (MNI ICBM152 1-mm 6th-generation atlas; [45]) using the AFNI function auto_warp.py. The transformation matrices obtained in the previous step were used to warp the BBB *k*_w_ maps to MNI152 space, using the AFNI function 3dNwarpApply and a wsinc5 cost function. The MNI152 warped BBB *k*_w_ images were used in subsequent voxel-wise analyses. Lastly, FreeSurfer 6.0 was used with the recon-all option to segment each participant’s *k*_w_ aligned T1-weighted image, in native space, into 89 cortical/subcortical ROIs (ROIs iterated in [53]). These 89 ROIs were eroded by one voxel to minimize partial volume effects and then resampled to the BBB *k*_w_ voxel resolution (i.e., from 1 mm^3^ isotropic to 3.5 × 3.5 × 8 mm^3^ voxels).

Average *k*_w_ values from the native space, *Z*-scored BBB *k*_w_ maps were then extracted from each of these resampled ROIs for each participant, under the constraint that at least 90% of the voxels in a given ROI overlapped with the BBB *k*_w_ map. This constraint is important because the BBB *k*_w_ maps did not cover the entire brain, whereas the T1-weighted images did. All 47 participants included in this study had greater than 90% coverage in all ROIs reported in subsequent sections. Extracted values were used in ROI-based analyses to more closely evaluate the relationship between BBB *k*_w_ and cerebrovascular dysfunction. Lastly, FreeSurfer estimated intracranial volumes (eICV, in mm^3^) were recorded for each participant. In all ROI-based regression analyses, eICV was included as a covariate to account for head size-dependent differences in ROI volumes across participants.

### Neuropsychological assessment

Forty-two out of the 47 participants of the cohort underwent neuropsychological testing using the National Alzheimer’s Coordinating Center’s (NACC) UDS-3 [42, 43]. The UDS-3 includes a comprehensive battery of neuropsychological tests assessing global cognition, memory encoding, memory retrieval, semantic memory, working memory, attention, executive function, processing speed, and verbal retrieval. Additional neuropsychological measures specific to the UK-ADRC were also administered to the participants, including the California verbal learning test, 2^nd^ edition (CVLT-II) used to construct the episodic memory composite scores described in the section below.

### Executive function (EF) and episodic memory (MEM) composite scores

Composite scores were created for the 42 participants who underwent neuropsychological testing, to assess EF and MEM. As such, all subsequent analyses that use these composite scores have a sample size of 42, whereas analyses that do not include these composite scores use the full participant cohort (*n* = 47). Better performance is reflected by higher composite scores for both EF and MEM. The EF composite scores were calculated using a validated software toolkit developed for the UDS3 [54] and scores from the following individual cognitive tests: category fluency (animals and vegetables), phonemic fluency (letter F and letter L), digit span backward, and trail making test A and B. Specifically, correct responses are used for category fluency, phonemic fluency, and digit span backward, whereas number of correct lines per minute are used for the trail making test.

The UDS-3 does not currently have a software toolkit for calculating MEM composite scores. As such, MEM composite scores were calculated using a factor score-based approach, comparable to the one used for the EF composite [54]. In more detail, individual test scores were first selected corresponding to those UDS-3/UK-ADC cognitive tests that best represent long-term memory performance. That is, those cognitive tests with the longest time delay between memory encoding and memory retrieval. These included the total story units recalled, using verbatim scoring from the Craft test, the delayed copy scores from the Benson complex figure test, and the long delay raw scores from the CVLT-II. An exploratory factor analysis indicated that the selected cognitive tests all represent a single latent factor (presumably long-term memory). Factor scores were then created for the single factor obtained in the previous step, using the Bartlett approach. This approach was selected because it provides 1) high validity (the factor scores are highly correlated to the estimated factor) and 2) unbiased estimates of factor score parameters [55].

### CVR imaging procedure

CVR was assessed using a previously described procedure [56–58]. Briefly, participants were fitted with a mouthpiece and nose-clip and mild hypercapnic air (5% carbon dioxide, 74% nitrogen, and 21% oxygen) was administered using a Douglas bag, with a two-way non-rebreathing valve, enabling precise switching between room-air and hypercapnic air [58]. Participants underwent blocked inhalation of hypercapnic air and room air while BOLD fMRI was acquired. The third author (C. B.) was present inside the scanner room throughout the experiment to manually switch the valve to control the breathing air type (either room air or hypercapnic air). The CVR paradigm used interleaved blocks comprised of 70 s room-air breathing and 50 s of hypercapnic air breathing, for a total scan time of 7.20 min. CO_2_ concentration in the exhaled air was sampled at 100 Hz, and the resulting CO_2_ trace was recorded using capnography (Philips Respironics NM3 Monitor, Model 7900, CT).

### CVR data processing

CVR analysis was performed using a cloud-based processing tool, CVR-MRICloud [38, 59] as follows: the BOLD data were first motion corrected and smoothed by an 8-mm Gaussian kernel using SPM12. The end-tidal CO_2_ (Et-CO_2_) was extracted from the CO_2_ trace using an algorithm to identify the peak CO_2_ of each exhaled breath and then the resulting Et-CO_2_ curve was temporally aligned (global-shifted) to the whole-brain BOLD signal time course. Unscaled, whole-brain CVR values were subsequently obtained using a general linear model (GLM) between whole-brain averaged BOLD signal and global-shifted EtCO2 (Eq. 1 in [38]). Lastly, the raw CVR values obtained in the previous step were scaled to units of %BOLD signal change (in the CO_2_-enhanced vs. normal air condition) per mm of mercury (Hg) of Et-CO_2_ change (%BOLD/mmHg CO_2_) using Eq. 2 in [38].

The resulting, individual participant CVR maps were then co-registered to their corresponding RMS T1-weighted images, which allowed warping/normalization of the CVR data to MNI space. In addition, the RMS T1-weighted images were segmented by the MultiAtlas Segmentation toolbox on MRICloud into 287 ROIs using the procedure described in [60, 61] and the “*Adult50-90_287Labels_30atlases_M2_252_V10A*” atlas. The segmented RMS T1 images were used to calculate per-ROI CVR values by constraining the whole-brain CVR procedure described previously within each ROI separately. That is, the Et-CO_2_ curve was temporally aligned to the BOLD signal time course within each ROI separately, followed by the GLM and scaling steps. Lastly, to provide an index of the overall quality of the CVR data, per ROI partial correlation coefficients were computed between the BOLD time course and temporally aligned EtCO_2_, after factoring out the linear drift. These correlation coefficients passed quality control (automatically evaluated by the CVR-MRICloud toolbox) in all ROIs reported in subsequent analyses.

### White matter hyperintensity volume quantification

WMHs were identified using a validated 4-tissue segmentation method [62] as follows. Participants’ FLAIR images were first registered to their corresponding RMS T1-weighted image using the FSL FLIRT function (FSL version 6.0.1; [63]), corrected for inhomogeneities using a previously published local histogram normalization [64], and then non-linearly warped to a standard atlas [62]. WMH probabilities were then estimated on the standard-atlas warped FLAIR images using a Bayesian probability structure, based on histogram fitting and prior probability maps [65]. The prior–probability maps were created from more than 700 individuals with semi-automatic detection of WMHs followed by manual editing [62]. The estimated WMH probabilities from the previous step were then thresholded at 3.5 SDs above the mean WM signal intensity of the FLAIR image to create per-participant, binary WMH masks. These binary WMH masks were then back-transformed to each participants’ native space FLAIR image. Lastly, the native space WMH masks were visually inspected and manually edited were necessary to assure quality.

Following visual inspection and manual editing of the WMH masks, ROI-based masking was used to split the native space WMH masks into periventricular (PV) and deep brain regions. PV WMHs were defined as being located within ~ 10 mm of the lateral ventricles and deep WMHs were defined as those located outside this radius [66]. The PV and deep ROIs used for masking were created using a validated group mask of the lateral ventricles in MNI152 space (MNI ICBM152 1-mm 6th-generation atlas; [45]), namely, the Automatic Lateral Ventricle delIneatioN (ALVIN) mask, created using data from 275 healthy adults (age range 18–94 [67];). The ALVIN mask extends beyond the lateral ventricles into brain parenchyma (~ 7–11 mm in most participants) to ensure inclusion of the lateral ventricles across participants with varying brain size. As such, the ALVIN mask, inversely warped from MNI152 space to each participant’s native space FLAIR image, can be used to delineate PV WMHs. Similarly, a whole-brain, binary mask in the same space that excludes the ALVIN mask can be used to delineate deep WMHs.

More specifically, the following procedure was used. First, each participant’s RMS T1 image was skull-stripped using FreeSurfer 6.0 and aligned to their corresponding FLAIR image in native space, using AFNIs align_epi_anat.py function and a local Pearson correlation cost function. The aligned and skull-stripped T1 images were then non-linearly warped to MNI152 space (MNI ICBM152 1-mm 6th-generation atlas; [45]) using the AFNI function auto_warp.py. The inverse of the transformation matrix obtained in the previous step was then used to inversely warp the ALVIN mask from MNI152 space to each participant’s FLAIR image in native space, using the AFNI function 3dNwarpApply and a nearest neighbor interpolation method. The inversely warped ALVIN masks were subsequently resampled to the FLAIR image grid using the 3dresample function in AFNI. Following the previous step, a binary brain mask was also created from the skull-stripped and FLAIR aligned RMS T1 images and resampled to the FLAIR image grid.

Each participant’s whole-brain WMH mask was then multiplied by their native-space ALVIN mask to create a PV WMH mask. Similarly, a deep WMH mask was created by multiplying each participant’s whole-brain WMH mask with a mask created by subtracting the native-space ALVIN mask from the FLAIR aligned and resampled, T1-based brain mask. Lastly, WMH volumes in mm^3^ were calculated for each participant by computing the number of voxels in the PV and deep WMH masks.

### Statistical analyses

Separate, group-level analyses were conducted with EF and MEM composite scores as independent variables (as mean centered vectors), and voxel-wise, *Z*-scored BBB *k*_w_ as the dependent variable, using linear mixed-effects models (3dLME; [68]). Covariates were age and years of education (as mean-centered vectors), and gender (as a categorical, between-subjects variable). The resulting statistical maps were thresholded at qFDR < 0.05 using the false discovery rate approach for multiple comparison correction [69].

To assess associations between BBB *k*_w_ and cerebrovascular dysfunction in brain regions where the voxel-wise analyses were significant, subsequent ROI-based analyses were conducted. These analyses focused on BBB *k*_*w*_ and CVR values extracted from those FreeSurfer and MRICloud/MultiAtlas ROIs that overlapped with significantly active voxels, as well as PV/deep WMH volumes. ROI analyses were then conducted in SPSS 27 (IBM, Chicago, IL, USA) using linear regression models with CVR values as the dependent variable and BBB *k*_w_ as the independent variable. Age, years of education, gender, and eICV values acted as covariates in all linear regression models.

For WMH volumes, two separate linear regression analyses were conducted, one for PV and another for deep WMH volumes. PV/deep WMH volumes acted as the dependent variable whereas average BBB *k*_w_ from all FreeSurfer ROIs that overlapped with significantly active voxels acted as independent variables. As in all previous linear regression models, age, years of education, gender, and eICV values were used as covariates. The WMH distributions were skewed as is typical and were log transformed. CVR values were also skewed and therefore also log-transformed in order to pass the Shapiro–Wilk test of normality [70]. All remaining variables were normally distributed. Variance inflation factors (VIF) are provided in all linear regression analyses in order to evaluate the degree of collinearity between independent variables. Multiple comparisons are reported using the Sidak correction unless stated otherwise.

## Results

### *Voxel-wise BBB k*_*w*_* vs. EF composite scores*

**Fig. 1:**
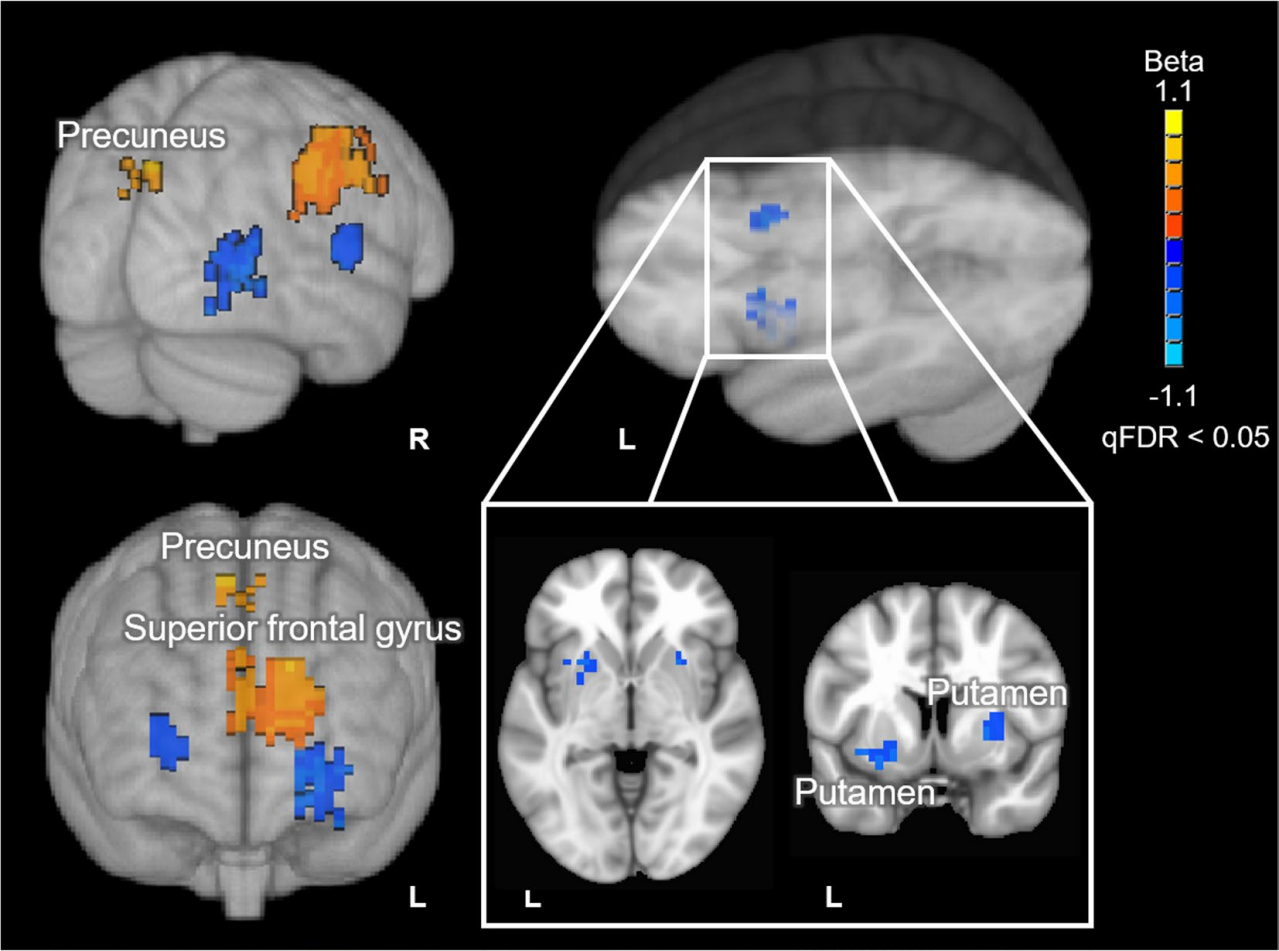
Voxel-wise results of the BBB *k*_w_ vs. EF composite score analysis. Positive correlations are depicted with warm (orange to yellow) colors and negative correlations are depicted with cool (blue to cyan) colors. Results are overlaid on the 1 mm, MNI152 template provided with FSL, rendered as a 3D volume. Notes: L = left hemisphere; R = right hemisphere

Results from this analysis revealed significant positive correlations between EF composite scores and BBB *k*_w_ in the left superior frontal gyrus and bilateral precuneus. In addition, negative correlations were observed between EF scores and BBB *k*_w_ in bilateral portions of the putamen (Fig. 1). The MNI152 coordinates of peak correlation voxels and their corresponding anatomical labels are provided in Table 2.

**Table 2:** MNI152 coordinates, cluster volume, and anatomical labels of voxels showing peak correlation between BBB *k*_w_ and EF composite scores

Anatomical brain region	Hemisphere^1^	Cluster volume (mm^3^)	MNI coordinates (*X*, *Y*, *Z*)^2^
Superior frontal gyrus	L	6689	− 12, 47, 25
Precuneus	R	429	3, − 83, 42
Putamen	L	2230	− 26, 8, − 11
Putamen	R	1544	28, 11,2

^1^Hemisphere (L: left; R: right)

^2^MNI coordinates, in LPI/SPM order

### *Voxel-wise BBB k*_*w*_* vs. MEM composite scores*

**Fig. 2:**
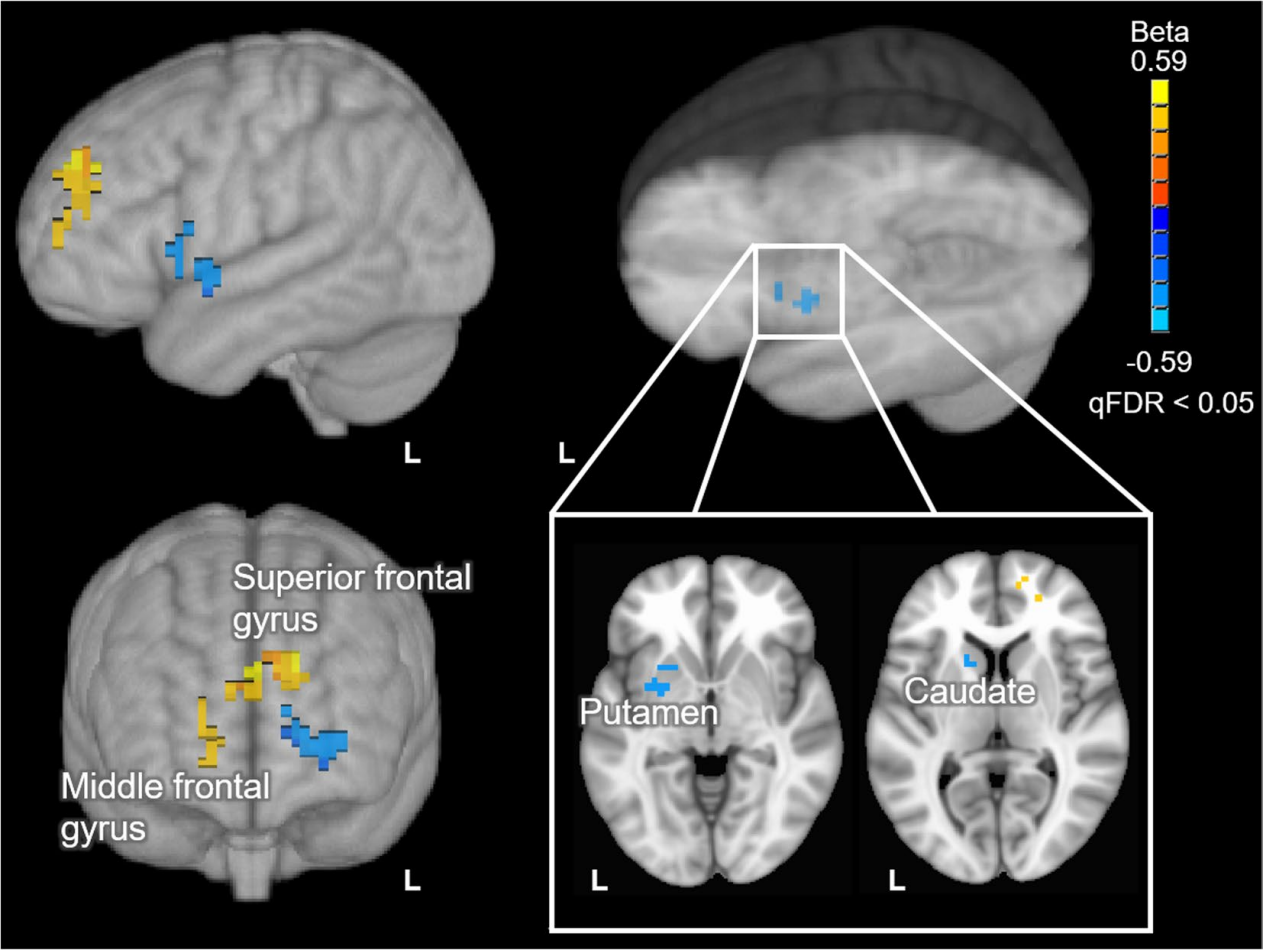
Voxel-wise results of the BBB *k*_w_ vs. MEM composite scores analysis. Positive correlations are depicted with warm (orange to yellow) colors and negative correlations are depicted with cool (blue to cyan) colors. Results are overlaid on the 1 mm, MNI152 template provided with FSL, rendered as a 3D volume. Notes: L = left hemisphere; R = right hemisphere

Results from this analysis revealed significant positive correlations between MEM composite scores and BBB *k*_w_ in the left superior and right middle frontal gyrus, as well as negative correlations between these scores and BBB *k*_w_ in the left caudate and putamen (Fig. 2). The MNI152 coordinates of peak correlation voxels and their corresponding anatomical label are provided in Table 3.

**Table 3:** MNI152 coordinates, cluster volume, and anatomical labels of voxels showing peak correlation between BBB *k*_w_ and MEM composite scores

Anatomical brain region	Hemisphere^1^	Cluster volume (mm^3^)	MNI coordinates (*X*, *Y*, *Z*)^2^
Superior frontal gyrus	L	1415	− 6, 51, 31
Middle frontal gyrus	R	557	17, 53, 10
Putamen	L	643	− 29, 2, − 4
Caudate	L	515	− 12, 8, 10

^1^Hemisphere (L: left; R: right)

^2^MNI coordinates, in LPI/SPM order

### *ROI-based analyses between BBB k*_*w*_* and CVR*

To evaluate potential links between BBB *k*_w_ within the brain regions identified in the voxel-wise analyses and MRI metrics of cerebrovascular function, linear regression analyses were first conducted between average BBB *k*_w_ and CVR extracted from the following GM ROIs: bilateral superior frontal gyrus, middle frontal gyrus, and precuneus. BBB *k*_w_ was also extracted from composite ROIs comprised of the bilateral caudate and putamen ROIs. All linear regression analyses controlled for age, years of education, gender, and eICV. Results are summarized in Table 4.

**Table 4:** Linear regression analyses results: BBB *k*_w_ vs. CVR in the superior frontal gyrus, middle frontal gyrus, precuneus, and basal ganglia ROIs. Results are adjusted for multiple comparisons using the Sidak correction such that *p* < 0.013 uncorrected corresponds to *p* < 0.05 corrected

ROI	^1^*β*	*r*^2^	*p*-value	SE	VIF	95% CI	
SFG	0.077	0.005	0.579	0.051	1.124	− 0.073	0.130
MFG	− 0.048	0.002	0.712	0.041	1.093	− 0.098	0.067
Precun	− 0.021	0.0004	0.865	0.026	1.063	− 0.056	0.048
BG	− 0.385	0.145	0.006*	0.026	1.053	− 0.126	− 0.022

Bold values indicate significant results

^1^Standardized coefficients

*SFG* superior frontal gyrus, *MFG* middle frontal gyrus, *Precun* precuneus, *BG* basal ganglia

*p < 0.05 Sidak corrected

No significant associations were identified between BBB *k*_w_ and CVR in the superior frontal gyrus, middle frontal gyrus, and precuneus ROIs. However, in the basal ganglia ROIs, a significant negative association between these measures was identified, indicating that higher BBB *k*_w_ was associated with lower CVR values.

### *ROI-based analyses between BBB k*_*w*_* and WMH volumes*

To further evaluate links between BBB *k*_w_ and vascular-related tissue damage, linear regression analyses were also conducted between average BBB *k*_w_ within the same ROIs used in the previous section and PV/deep WMH volumes, while controlling for age, years of education, gender, and eICV. Results are summarized in Table 5. A single significant, positive association was observed between PV WMH volumes and BBB *k*_w_ within the basal ganglia ROIs, such that higher BBB *k*_w_ was associated with larger PV WMH volumes.

**Table 5:** Linear regression analyses results: BBB *k*_w_ in the superior frontal gyrus, middle frontal gyrus, precuneus, and basal ganglia ROIs vs. PV/deep WMH volume (mm^3^). Results are adjusted for multiple comparisons using the Sidak correction such that *p* < 0.025 uncorrected corresponds to *p* < 0.05 corrected

PV WMH volume							
ROI	^1^*β*	*r*^2^	*p*-value	SE	VIF	95% CI	
SFG	0.088	0.010	0.389	0.364	1.202	− 0.410	1.040
MFG	− 0.011	0.002	0.911	0.311	1.192	− 0.654	0.585
Precun	− 0.052	0.006	0.584	0.185	1.038	− 0.470	0.267
BG	0.218	0.060	0.024*	0.172	1.066	0.050	0.734
Deep WMH volume							
ROI	^1^*β*	*r*^2^	*p*-value	SE	VIF	95% CI	
SFG	0.171	0.013	0.121	0.632	1.202	− 0.266	2.254
MFG	− 0.025	0.003	0.821	0.540	1.192	− 1.199	0.954
Precun	− 0.092	0.027	0.366	0.321	1.038	− 0.933	0.348
BG	0.161	0.023	0.119	0.298	1.066	− 0.123	1.066

Bold values indicate significant results

^1^Standardized coefficients

*SFG* superior frontal gyrus, *MFG* middle frontal gyrus, *Precun* precuneus, *BG* basal ganglia, *PV* periventricular

*p < 0.05 Sidak corrected

### ROI-based analyses between CVR, PV, and WMH volumes and cognitive performance in the basal ganglia

A significant negative association was found between BBB *k*_w_ and CVR in the basal ganglia, while a positive association was observed between BBB *k*_w_ in the same region and PV WMH volumes. These findings raise the question of whether CVR values in the basal ganglia and neighboring PV WMH volumes also correlate significantly with EF and MEM composite scores, suggesting possible mediation effects of cerebrovascular dysfunction or vascular-related tissue damage on the association between BBB *k*_w_ and cognitive performance in the basal ganglia. To test this possibility, two separate linear regression analyses were conducted, one with EF and another with MEM as the dependent variable and average basal ganglia CVR values and PV WMH volumes as independent variables in the same model. Age, years of education, gender, and eICV were added as covariates in both regression models. Results are summarized in Table 6. A significant positive correlation was identified between CVR in the basal ganglia and EF; however, PV WMH did not correlate significantly with EF. Neither CVR nor PV WMH volumes correlated significantly with MEM.

**Table 6:** Linear regression analyses results: basal ganglia CVR and PV WMH volumes vs. EF and MEM composite scores. Results are adjusted for multiple comparisons using the Sidak correction such that *p* < 0.025 uncorrected corresponds to *p* < 0.05 corrected

EF							
	^1^*β*	*r*^2^	*p*-value	SE	VIF	95% CI	
PV WMH	− 0.220	0.055	0.168	0.121	1.47	− 0.416	0.076
CVR	0.344	0.161	0.015*	0.961	1.09	0.504	4.410
MEM							
	^1^*β*	*r*^2^	*p*-value	SE	VIF	95% CI	
PV WMH	− 0.274	0.064	0.136	0.145	1.47	− 0.517	0.073
CVR	0.229	0.061	0.147	1.154	1.09	− 0.631	4.061

Bold values indicate significant results

^1^Standardized coefficients

*p < 0.05 Sidak corrected

### *The effect of CVR on the association between BBB k*_*w*_* and cognitive performance in the basal ganglia*

A significant negative association was found between BBB *k*_w_ and CVR in the basal ganglia, while a positive association was observed between CVR in this region and EF. This raises the question of whether the BBB *k*_w_–EF associations we observed in the voxel-wise analyses are driven by alterations in the transport of water across the BBB per se, or more general vascular dysfunction associated with CVR. To test between these alternatives, a mediation analysis was conducted, testing the impact of CVR as a mediator on the direct effect of BBB *k*_w_ on EF. The mediation analysis was conducted in SPSS using the Andrew F. Hayes PROCESS (version 3.5; model 4) computational tool [71]. The mediation results were evaluated at 95% confidence intervals using the default setting of 5000 bootstrapped samples. As in all previous analyses, age, years of education, gender, and eICV were added as covariates. The results are presented in Table 7.

**Table 7:** Mediation analysis results (process 3.5): basal ganglia CVR as a mediator of the direct effect of BBB *k*_w_ on EF composite scores

	Effect	SE	t	p	95% CI	
Direct effect of *k*_w_ on EF	− 0.536	0.234	− 2.29	0.031*	− 1.0196	− 0.0527
Indirect effect of CVR on *k*_w_ vs. EF	− 0.020	^1^0.095	**-**	**-**	− 0.2854	0.1048

Bold values indicate significant results

^1^Bootstrapped SE

*EF* executive function composite scores

*p < 0.05

As anticipated from the voxel-wise results presented previously, a negative direct effect was observed between BBB *k*_w_ in the basal ganglia and EF composite scores (Table 7). However, CVR did not mediate the direct effect of BBB *k*_w_ on EF.

## Discussion

Our results revealed significant correlations between BBB *k*_w_ and both executive function and episodic memory performance. However, the direction of these associations varied across brain regions. Furthermore, BBB *k*_w_ in the basal ganglia, but not in frontoparietal brain regions, was negatively associated with CVR and positively associated with periventricular WMH volume. Our findings collectively indicate that BBB *k*_w_ is a sensitive metric of cognitive function in older adults, but appears to show different directions of association in neocortical and basal ganglia regions. These regionallydependent associations may relate to different physiological underpinnings in the relationships between BBB *k*_w_ and cognition in neocortical versus subcortical brain regions in older adults.

### *BBB k*_*w*_* and cognitive performance*

Our voxel-wise results present novel evidence linking BBB *k*_w_ to cognitive performance in task relevant brain regions in older adults, after controlling for age, gender, years of education, and eICV. Specifically, BBB *k*_w_ in the frontal and parietal lobe brain regions and in the putamen was associated with executive function, while BBB *k*_w_ in the frontal lobe brain regions, caudate, and putamen was associated with episodic memory. Frontoparietal cortical brain regions are consistently implicated in executive functions, such as cognitive control [72–74], and frontal lobe regions are known to play a role in episodic memory retrieval [75–78].

The basal ganglia is strongly associated with initiation of movement [79] and motor learning [80], but the dorsal striatum (caudate and putamen) of the basal ganglia also play key roles in higher cognitive functions such as executive function and episodic memory. For example, the caudate and putamen have been associated with planning and set-shifting, the ability to alter a response in the face of changing circumstances [81], and other forms of cognitive control [82]. Additionally, the putamen has been linked to verbal episodic memory, particularly with performance on the CVLT-II [83], which is one of the cognitive tests used to create the episodic memory composite scores in the current study.

### *BBB k*_*w*_* and cognitive performance: direction of associations*

 The direction of the *k*_w_–cognition associations we observed varied across brain regions, with positive associations observed in the frontal and parietal brain regions and negative associations observed in basal ganglia regions. The positive associations observed between BBB *k*_w_ and cognitive performance in frontal and parietal brain regions are in keeping with the BBB *k*_*w*_–cognitive performance trends previously reported by our group in the same cortical brain regions, in a cohort of cognitively normal older adults [33]. Moreover, similar associations in the neocortical brain regions have been reported by other researchers exploring BBB *k*_w_ in patient populations [31, 32, 84]. Results from the present study provide further evidence that high BBB *k*_w_ in the neocortical regions is associated with high cognitive performance.

In contrast to the positive associations observed between BBB *k*_w_ and cognition in the frontoparietal regions, the present study observed negative associations between BBB *k*_w_ and cognition in the basal ganglia. The basal ganglia is a region that is particularly susceptible to age-related vascular dysfunction and degradation [85–88]. Specifically, the sole blood supply to the basal ganglia and neighboring WM comes from the lenticulostriate arteries (LSAs) [89], which are small and tortuous with no collaterals, rendering them more susceptible to flow disruption caused by arteriosclerosis and/or ventricular expansion associated with aging [90]. Therefore, we considered the possibility that the negative association between BBB *k*_w_ and cognition in the basal ganglia may be related to vascular dysfunction and/or vascular-related tissue damage.

Our results indicated a selective negative association between BBB *k*_w_ and CVR in the basal ganglia, with no comparable association in frontoparietal regions. Similarly, a selective positive association was observed between BBB *k*_w_ in the basal ganglia and PV WMH volumes that was not present in frontoparietal regions. Notably, this positive association in the basal ganglia was only significant for regional, PV WMH volumes and not more distal deep WMH volumes. PV WMHs, the most prevalent type of WMHs [90], are believed to be in part a consequence of vascular damage to the LSAs. Thus, there is an established connection between the basal ganglia and neighboring PV WMHs in terms of the LSA blood supply, contributing to their common susceptibility to age-related vascular dysfunction and degradation*.* Importantly, this arterial supply is not shared by deeper WM regions, which are not supplied by the LSAs. Therefore, the selective positive association we observed between BBB *k*_w_ in the basal ganglia and PV WMH volumes only (not deep WMHs) is 1) consistent with the basal ganglia being a region where vascular dysfunction and/or damage tends to be prominent and 2) suggests a link between *k*_w_ in the basal ganglia and neighboring vascular-related structural tissue damage.

### *Potential physiological underpinnings of the BBB k*_*w*_*–cognition associations in neocortex and in the basal ganglia*

In our previous study, we found that high BBB *k*_w_ in neocortical regions, but not hippocampus, was associated with high concentrations of amyloid beta (Aβ)42 in cerebrospinal fluid (CSF; indicative of low Aβ deposition in neuritic plaques). Thus, our previous results suggested that high BBB *k*_w_ values in neocortical regions may be indicative of increased BBB Aβ clearance rates in older adults [33]. Other studies exploring BBB *k*_w_ in patient populations have adopted similar conclusions, linking high *k*_w_ to optimal BBB clearance functions [31, 32]. Optimal Aβ clearance rates are likely to be of most relevance to cognition, at least in healthy older adults, when considering neocortical regions, as Aβ deposition begins in neocortex [91, 92]. Thus, the positive associations we observed between *k*_w_ and cognition in frontoparietal regions in this study are not inconsistent with an optimal clearance function explanation.

In contrast, the basal ganglia is not an early site of common proteinopathies such as Aβ deposition [91, 92] or Lewy body deposition (comprising aggregates of ubiquitin and alpha-synuclein) [93]. However, the basal ganglia is a major site of age-related cSVD, which could contribute to the negative relationship between BBB *k*_w_ and cognition we observed in this region. Indeed, the significant links between BBB *k*_w_ in the basal ganglia, CVR, and PV WMH volumes we observed here suggest that the association between BBB *k*_w_ and cognition in this region may be related to vascular dysfunction and/or damage. Notably, however, the negative association between BBB *k*_w_ and cognitive performance was not mediated by regional CVR or neighboring PV WMH volumes. This latter finding suggests that BBB *k*_w_ in the basal ganglia may be uniquely sensitive to abnormal BBB water exchange that is associated with but extends beyond general cerebrovascular dysfunction/damage.

One possibility is that age-related arterial stiffening and associated vascular degradation in the basal ganglia may trigger the overexpression of perivascular water channels, leading to higher BBB water permeability and increased BBB *k*_w_. The water channel protein aquaporin allows water molecules to pass through the BBB one at a time [94]. This limits the rate of water exchange across the BBB [22, 95–97], which is essential for protecting the brain from swelling and edema [21, 25]. Vascular degradation can trigger overexpression of these channels [98–100], increasing the BBB’s water permeability and leading to reduced cognitive performance. We also cannot eliminate the possibility that the negative association between BBB *k*_w_ and cognition in the basal ganglia could in part reflect loss of BBB integrity or BBB leakage, for example, age-related endothelial degeneration leading to loss of tight junctions [101]. To evaluate this possibility, biofluid markers of BBB integrity are necessary, such as measures of CSF/plasma albumin ratio [5, 10, 102]. This possibility will be explored in a future study.

Finally, it should be noted that some of our findings differ from those observed by [32]. Specifically, [32] found negative associations between BBB *k*_w_ and markers of cSVD in the whole brain, temporal lobe, normal appearing WM, and putamen, in patients with cerebral autosomal dominant arteriopathy with subcortical infarcts and leukoencephalopathy (CADASIL). In contrast, in our study, we found positive associations between BBB *k*_w_ and cSVD measures in the caudate and putamen and no relationship between BBB k_w_ and measures of cSVD in neocortical regions. This divergence may be attributed to reduced expression of the water channel protein aquaporin-4 (AQP4) in the BBB of CADASIL patients, leading to low BBB *k*_w_ values. Previous pathological studies have shown that reduced AQP4 expression in the BBB is a characteristic of CADASIL, associated with both chronic hypoperfusion of brain tissue and *NOTCH3* gene mutations which can lead to apoptosis of astrocytes and increased clasmatodendritic astrocytes with displaced AQP4 [103–105]. It should also be noted that CADASIL and other cerebrovascular disorders may prolong arterial/arteriolar transit times which may contribute to lower BBB *k*_w_ values.

### Limitations

This was a cross-sectional study that is limited to describing correlations. Future longitudinal studies are necessary to evaluate causal relationships between BBB *k*_w_, cognitive ability, and cerebrovascular dysfunction in normal aging. Further, while this study provides evidence for different physiological underpinnings underlying the relationship between BBB *k*_w_ and cognition in cortical versus subcortical brain regions, the specific mechanisms driving these associations require further investigation. Animal model physiological and/or histological validation studies of BBB *k*_w_ are necessary to directly test specific biological mechanisms linking BBB function and/or degradation to the *k*_w_ metric. The use of other independent water permeability techniques may also be helpful in further elucidating the role of BBB alteration in cognitive aging [106–108]. Lastly, the current study employed a DP-ASL sequence with relatively low-resolution to assess BBB *k*_w_. The use of a relatively low-resolution DP-ASL sequence limits our ability to investigate potential lateralization effects in the relationship between *k*_w_ and cognition, particularly affecting voxel clusters near the midline. Future research using higher-resolution DP-ASL sequences [109] will be better positioned to investigate potential lateralization effects.

## Conclusions

The present study provides novel evidence suggesting that the non-invasive, MRI measure of BBB *k*_w_ is associated with cognitive functioning in healthy older adults. Further, the current study provides preliminary evidence suggesting that BBB *k*_w_ may be associated with different physiological underpinnings in neocortex compared to subcortical structures. In particular, *k*_w_ in the basal ganglia maybe sensitive to alterations in the transport of water across the BBB that are associated with, but extend beyond, general cerebrovascular dysfunction. Future studies should more thoroughly explore the relationship between BBB *k*_w_ and cerebrovascular dysfunction in the subcortical brain regions using biofluid markers of BBB integrity.

## Data Availability

The raw data supporting the conclusions of this article will be made available by the authors, without undue reservation.

## References

[1] Zimmerman B, Rypma B, Gratton G, Fabiani M (2021). Age-related changes in cerebrovascular health and their effects on neural function and cognition: a comprehensive review. Psychophysiology.

[2] Camici GG, Liberale L (2017). Aging: the next cardiovascular disease?. Eur Heart J.

[3] Yang T, Sun Y, Lu Z, Leak RK, Zhang F (2017). The impact of cerebrovascular aging on vascular cognitive impairment and dementia. Ageing Res Rev.

[4] Marchant NL, Reed BR, DeCarli CS, Madison CM, Weiner MW, Chui HC (2012). Cerebrovascular disease, beta-amyloid, and cognition in aging. Neurobiol Aging.

[5] Montagne A, Barnes SR, Sweeney MD, Halliday MR, Sagare AP, Zhao Z (2015). Blood-brain barrier breakdown in the aging human hippocampus. Neuron.

[6] Senatorov VV, Friedman AR, Milikovsky DZ, Ofer J, Saar-Ashkenazy R, Charbash A (2019). Blood-brain barrier dysfunction in aging induces hyperactivation of TGFβ signaling and chronic yet reversible neural dysfunction. Sci Transl Med.

[7] Nation DA, Sweeney MD, Montagne A, Sagare AP, D’Orazio LM, Pachicano M (2019). Blood–brain barrier breakdown is an early biomarker of human cognitive dysfunction. Nat Med.

[8] Farrall AJ, Wardlaw JM (2009). Blood-brain barrier: ageing and microvascular disease–systematic review and meta-analysis. Neurobiol Aging.

[9] Shah GN, Mooradian AD (1997). Age-related changes in the blood-brain barrier. Exp Gerontol.

[10] Lin Z, Sur S, Liu P, Li Y, Jiang D, Hou X (2021). Blood–brain barrier breakdown in relationship to alzheimer and vascular disease. Ann Neurol.

[11] Abbott NJ, Patabendige AAK, Dolman DEM, Yusof SR, Begley DJ (2010). Structure and function of the blood–brain barrier. Neurobiol Dis.

[12] Galea I (2021). The blood–brain barrier in systemic infection and inflammation. Cell Mol Immunol.

[13] Zlokovic BV (2008). The blood-brain barrier in health and chronic neurodegenerative disorders. Neuron.

[14] Daneman R (2012). The blood–brain barrier in health and disease. Ann Neurol.

[15] Verheggen ICM, de Jong JJA, van Boxtel MPJ, Gronenschild EHBM, Palm WM, Postma AA (2020). Increase in blood–brain barrier leakage in healthy, older adults. GeroScience.

[16] Manning C, Stringer M, Dickie B, Clancy U, Valdés Hernandez MC, Wiseman SJ (2021). Sources of systematic error in DCE-MRI estimation of low-level blood-brain barrier leakage. Magn Reson Med.

[17] Montagne A, Barnes SR, Nation DA, Kisler K, Toga AW, Zlokovic BV (2022). Imaging subtle leaks in the blood–brain barrier in the aging human brain: potential pitfalls, challenges, and possible solutions. GeroScience.

[18] Li M, Li Y, Zuo L, Hu W, Jiang T (2021). Increase of blood-brain barrier leakage is related to cognitive decline in vascular mild cognitive impairment. BMC Neurol.

[19] Starr JM, Farrall AJ, Armitage P, McGurn B, Wardlaw J (2009). Blood–brain barrier permeability in Alzheimer’s disease: a case–control MRI study. Psychiatry Res Neuroimaging.

[20] Barnes SR, Ng TSC, Montagne A, Law M, Zlokovic BV, Jacobs RE (2016). Optimal acquisition and modeling parameters for accurate assessment of low Ktrans blood–brain barrier permeability using dynamic contrast-enhanced MRI. Magn Reson Med.

[21] Shao X, Ma SJ, Casey M, D’Orazio L, Ringman JM, Wang DJJ (2019). Mapping water exchange across the blood–brain barrier using 3D diffusion-prepared arterial spin labeled perfusion MRI. Magn Reson Med.

[22] Wang J, Fernández-Seara MA, Wang S, St Lawrence KS (2007). When perfusion meets diffusion: in vivo measurement of water permeability in human brain. J Cereb Blood Flow Metab.

[23] St. Lawrence KS, Owen D, Wang DJJ (2012). A two-stage approach for measuring vascular water exchange and arterial transit time by diffusion-weighted perfusion MRI. Magn Reson Med.

[24] Agre P, King LS, Yasui M, Guggino WB, Ottersen OP, Fujiyoshi Y (2002). Aquaporin water channels–from atomic structure to clinical medicine. J Physiol.

[25] Agre P (2006). The aquaporin water channels. Proc Am Thorac Soc.

[26] Papadopoulos MC, Manley GT, Krishna S, Verkman AS (2004). Aquaporin-4 facilitates reabsorption of excess fluid in vasogenic brain edema. FASEB J.

[27] Vella J, Zammit C, Di Giovanni G, Muscat R, Valentino M. The central role of aquaporins in the pathophysiology of ischemic stroke. Front Cell Neurosci. 2015;9(APR). 10.3389/FNCEL.2015.00108 10.3389/fncel.2015.00108 PMC4389728 25904843

[28] Silva AC, Williams DS, Koretsky AP (1997). Evidence for the exchange of arterial spin-labeled water with tissue water in rat brain from diffusion-sensitized measurements of perfusion. Magn Reson Med.

[29] Dickie BR, Parker GJM, Parkes LM (2020). Measuring water exchange across the blood-brain barrier using MRI. Prog Nucl Magn Reson Spectrosc.

[30] Tiwari YV, Lu J, Shen Q, Cerqueira B, Duong TQ (2017). Magnetic resonance imaging of blood–brain barrier permeability in ischemic stroke using diffusion-weighted arterial spin labeling in rats. J Cereb Blood Flow Metab.

[31] Uchida Y, Kan H, Sakurai K, Horimoto Y, Hayashi E, Iida A (2022). APOE ɛ4 dose associates with increased brain iron and β-amyloid via blood–brain barrier dysfunction. J Neurol Neurosurg Psychiatry.

[32] Li Y, Ying Y, Yao T, Jia X, Liang H, Tang W (2023). Decreased water exchange rate across blood–brain barrier in hereditary cerebral small vessel disease. Brain.

[33] Gold BT, Shao X, Sudduth TL, Jicha GA, Wilcock DM, Seago ER (2021). Water exchange rate across the blood-brain barrier is associated with CSF amyloid-β 42 in healthy older adults. Alzheimer’s Dement.

[34] Harada CN, Natelson Love MC, Triebel KL (2013). Normal cognitive aging. Clin Geriatr Med.

[35] Blacker D, Lee H, Muzikansky A, Martin EC, Tanzi R, McArdle JJ (2007). Neuropsychological measures in normal individuals that predict subsequent cognitive decline. Arch Neurol.

[36] Salthouse TA (2009). When does age-related cognitive decline begin?. Neurobiol Aging.

[37] Liu P, De Vis JB, Lu H (2019). Cerebrovascular reactivity (CVR) MRI with CO2 challenge: a technical review. Neuroimage.

[38] Liu P, Baker Z, Li Y, Li Y, Xu J, Park DC (2022). CVR-MRICloud: an online processing tool for CO2-inhalation and resting-state cerebrovascular reactivity (CVR) MRI data. PLoS One.

[39] Erten-Lyons D, Woltjer R, Kaye J, Mattek N, Dodge HH, Green S (2013). Neuropathologic basis of white matter hyperintensity accumulation with advanced age. Neurology.

[40] Wardlaw JM, Valdés Hernández MC, Muñoz-Maniega S (2015). What are white matter hyperintensities made of?. J Am Heart Assoc.

[41] Schmitt AF, Nelson TP, Abner E, Scheff S, Jicha AG, Smith C (2013). University of Kentucky Sanders-Brown healthy brain aging volunteers: donor characteristics, procedures and neuropathology. Curr Alzheimer Res.

[42] Morris JC, Weintraub S, Chui HC, Cummings J, DeCarli C, Ferris S (2006). The uniform data set (UDS): clinical and cognitive variables and descriptive data from Alzheimer Disease Centers. Alzheimer Dis Assoc Disord.

[43] Besser L, Kukull W, Knopman DS, Chui H, Galasko D, Weintraub S (2018). Version 3 of the national Alzheimer’s coordinating center’s uniform data set. Alzheimer Dis Assoc Disord.

[44] Nasreddine ZS, Phillips NA, BÃ©dirian V, Charbonneau S, Whitehead V, Collin I (2005). The Montreal Cognitive Assessment, MoCA: a brief screening tool for mild cognitive impairment. J Am Geriatr Soc.

[45] Grabner G, Janke AL, Budge MM, Smith D, Pruessner J, Collins DL. Symmetric atlasing and model based segmentation: an application to the hippocampus in older adults. Lecture Notes in Computer Science (including subseries Lecture Notes in Artificial Intelligence and Lecture Notes in Bioinformatics). Springer Verlag; 2006; 58–66. 10.1007/11866763_8 17354756

[46] van der Kouwe AJW, Benner T, Salat DH, Fischl B (2008). Brain morphometry with multiecho MPRAGE. Neuroimage.

[47] Shao X, Wang Y, Moeller S, Wang DJJ (2018). A constrained slice-dependent background suppression scheme for simultaneous multislice pseudo-continuous arterial spin labeling. Magn Reson Med.

[48] Penny W, Friston K, Ashburner J, Kiebel S, Nichols T (2007). Statistical parametric mapping: the analysis of functional brain images. Stat Parametr Mapp Anal Funct Brain Images.

[49] Shao X, Dylan Tisdall M, Wang DJ, Jan A, Van Der Kouwe W. Prospective motion correction for 3D GRASE pCASL with volumetric navigators. Proc Int Soc Magn Reson Med Sci Meet Exhib Int Soc Magn Reson Med Sci Meet Exhib. 2017 Apr [cited 2023 Feb 15]. ;25:0680. PMC5891141 29643745

[50] Spann SM, Shao X, Wang DJ, Aigner CS, Schloegl M, Bredies K (2020). Robust single-shot acquisition of high resolution whole brain ASL images by combining time-dependent 2D CAPIRINHA sampling with spatio-temporal TGV reconstruction. Neuroimage.

[51] Lu H, Clingman C (2004). Golay X, Zijl PCM van. Determining the longitudinal relaxation time (T1) of blood at 3.0 Tesla. Magn Reson Med.

[52] Cox RW. AFNI: software for analysis and visualization of functional magnetic resonance neuroimages. Comput Biomed Res. 1996:29(3):162–173. 10.1006/cbmr.1996.0014 8812068

[53] Zachariou V, Bauer CE, Powell DK, Gold BT (2022). Ironsmith: an automated pipeline for QSM-based data analyses. Neuroimage.

[54] Staffaroni AM, Asken BM, Casaletto KB, Fonseca C, You M, Rosen HJ (2021). Development and validation of the Uniform Data Set (v.30) executive function composite score (UDS3-EF). Alzheimer’s Dement.

[55] Hershberger SL (2005). Factor score estimation. Encycl Stat Behav Sci.

[56] Liu P, Jiang D, Albert M, Bauer CE, Caprihan A, Gold BT (2021). Multi-vendor and multisite evaluation of cerebrovascular reactivity mapping using hypercapnia challenge. Neuroimage.

[57] Lu H, Liu P, Yezhuvath U, Cheng Y, Marshall O, Ge Y. MRI mapping of cerebrovascular reactivity via gas inhalation challenges. JoVE (Journal Vis Exp. 2014;(94):e52306. 10.3791/52306 PMC4396915 25549106

[58] Lu H, Kashani AH, Arfanakis K, Caprihan A, DeCarli C, Gold BT (2021). MarkVCID Cerebral small vessel consortium: II Neuroimaging protocols. Alzheimers Dement.

[59] Mori S, Wu D, Ceritoglu C, Li Y, Kolasny A, Vaillant MA (2016). MRICloud: delivering high-throughput MRI neuroinformatics as cloud-based software as a service. Comput Sci Eng.

[60] Wang H, Pouch A, Takabe M, Jackson B, Gorman J, Gorman R (2013). Multi-atlas segmentation with robust label transfer and label fusion. Lect Notes Comput Sci (including Subser Lect Notes Artif Intell Lect Notes Bioinformatics).

[61] Tang X, Oishi K, Faria AV, Hillis AE, Albert MS, Mori S (2013). Bayesian parameter estimation and segmentation in the multi-atlas random orbit model. PLoS ONE.

[62] DeCarli C., Maillard P., Fletcher E. (2013). Four tissue segmentation in ADNI II. Alzheimer's Disease neuroimaging initiative. Available online at: https://files.alz.washington.edu/documentation/adni-proto.pdf

[63] Jenkinson M, Beckmann CF, Behrens TEJ, Woolrich MW, Smith SM (2012). FSL. Neuroimage.

[64] DeCarli C, Murphy DGM, Teichberg D, Campbell G, Sobering GS (1996). Local histogram correction of MRI spatially dependent image pixel intensity nonuniformity. J Magn Reson Imaging.

[65] DeCarli C, Miller BL, Swan GE, Reed T, Wolf PA, Garner J (1999). Predictors of brain morphology for the men of the NHLBI twin study. Stroke.

[66] Griffanti L, Jenkinson M, Suri S, Zsoldos E, Mahmood A, Filippini N (2018). Classification and characterization of periventricular and deep white matter hyperintensities on MRI: a study in older adults. Neuroimage.

[67] Kempton MJ, Underwood TSA, Brunton S, Stylios F, Schmechtig A, Ettinger U (2011). A comprehensive testing protocol for MRI neuroanatomical segmentation techniques: evaluation of a novel lateral ventricle segmentation method. Neuroimage.

[68] Chen G, Saad ZS, Britton JC, Pine DS, Cox RW (2013). Linear mixed-effects modeling approach to FMRI group analysis. Neuroimage.

[69] Genovese CR, Lazar NA, Nichols T (2002). Thresholding of statistical maps in functional neuroimaging using the false discovery rate. Neuroimage.

[70] Shapiro SS, Wilk MB (1965). An analysis of variance test for normality (complete samples). Biometrika.

[71] Hayes AF. Introduction to mediation, moderation, and conditional process analysis. Guilford Press; 2019; [cited 2022 Aug 26].Available from: https://www.scirp.org/(S(351jmbntvnsjt1aadkozje))/reference/ReferencesPapers.aspx?ReferenceID=2277421

[72] Yeager BE, Bruss J, Duffau H, Herbet G, Hwang K, Tranel D (2022). Central precuneus lesions are associated with impaired executive function. Brain Struct Funct.

[73] Menon V, D’Esposito M (2021). The role of PFC networks in cognitive control and executive function. Neuropsychopharmacol.

[74] Kim C, Johnson NF, Gold BT (2012). Common and distinct neural mechanisms of attentional switching and response conflict. Brain Res.

[75] Lepage M, Ghaffar O, Nyberg L, Tulving E (2000). Prefrontal cortex and episodic memory retrieval mode. Proc Natl Acad Sci.

[76] Shallice T, Fletcher P, Frith CD, Grasby P, Frackowiak RSJ, Dolan RJ (1994). Brain regions associated with acquisition and retrieval of verbal episodic memory. Nat.

[77] Epelbaum S, Bouteloup V, Mangin JF, La Corte V, Migliaccio R, Bertin H (2018). Neural correlates of episodic memory in the Memento cohort. Alzheimer’s Dement Transl Res Clin Interv.

[78] Wiggs CL, Weisberg J, Martin A (1998). Neural correlates of semantic and episodic memory retrieval. Neuropsychologia.

[79] Wichmann T, Delong MR (1996). Functional and pathophysiological models of the basal ganglia. Curr Opin Neurobiol.

[80] Doyon J, Benali H (2005). Reorganization and plasticity in the adult brain during learning of motor skills. Curr Opin Neurobiol.

[81] Monchi O, Petrides M, Strafella AP, Worsley KJ, Doyon J (2006). Functional role of the basal ganglia in the planning and execution of actions. Ann Neurol.

[82] Pauli WM, O’Reilly RC, Yarkoni T, Wager TD (2016). Regional specialization within the human striatum for diverse psychological functions. Proc Natl Acad Sci U S A.

[83] Ystad M, Eichele T, Lundervold AJ, Lundervold A (2010). Subcortical functional connectivity and verbal episodic memory in healthy elderly—a resting state fMRI study. Neuroimage.

[84] Goldwaser EL, Wang DJJ, Adhikari BM, Chiappelli J, Shao X, Yu J (2023). Evidence of Neurovascular water exchange and endothelial vascular dysfunction in schizophrenia: an exploratory study. Schizophr Bull.

[85] Bown CW, Carare RO, Schrag MS, Jefferson AL (2022). Physiology and clinical relevance of enlarged perivascular spaces in the aging brain. Neurology.

[86] Perosa V, Arts T, Assmann A, Mattern H, Speck O, Oltmer J (2022). Pulsatility index in the basal ganglia arteries increases with age in elderly with and without cerebral small vessel disease. Am J Neuroradiol.

[87] Rivera-Rivera LA, Schubert T, Turski P, Johnson KM, Berman SE, Rowley HA (2017). Changes in intracranial venous blood flow and pulsatility in Alzheimer’s disease: a 4D flow MRI study. J Cereb Blood Flow Metab.

[88] Uehara T, Tabuchi M, Mori E (1999). Risk factors for silent cerebral infarcts in subcortical white matter and basal ganglia. Stroke.

[89] Xu X, Wu X, Zhu C, Zhang R, Jiaerken Y, Wang S, et al. Characterization of lenticulostriate arteries and its associations with vascular risk factors in community-dwelling elderly. Front Aging Neurosci. 2021;13. 10.3389/FNAGI.2021.685571 10.3389/fnagi.2021.685571 PMC8258402 34239436

[90] Wen W, Sachdev P (2004). The topography of white matter hyperintensities on brain MRI in healthy 60- to 64-year-old individuals. Neuroimage.

[91] Braak H, Braak E (1991). Neuropathological stageing of Alzheimer-related changes. Acta Neuropathol.

[92] Thal DR, Rüb U, Orantes M, Braak H (2002). Phases of Aβ-deposition in the human brain and its relevance for the development of AD. Neurology.

[93] Rezaie P, Cairns NJ, Chadwick A, Lantos PL (1996). Lewy bodies are located preferentially in limbic areas in diffuse Lewy body disease. Neurosci Lett.

[94] Agre P (2005). Aquaporin water channels. Biosci Rep.

[95] St Lawrence KS, Frank JA, McLaughlin AC (2000). Effect of restricted water exchange on cerebral blood flow values calculated with arterial spin tagging: a theoretical investigation. Magn Reson Med.

[96] Paulson OB (2002). Blood–brain barrier, brain metabolism and cerebral blood flow. Eur Neuropsychopharmacol.

[97] Herscovitch P, Raichle ME, Kilbourn MR, Welch MJ. Positron emission tomographic measurement of cerebral blood flow and permeability—surface area product of water using [15O]water and [11C]butanol. http://dx.doi.org.ezproxy.uky.edu/101038/jcbfm1987102. 1987 Oct;7(5):527–42. 10.1038/jcbfm.1987.102 3498732

[98] Suzuki R, Okuda M, Asai J, Nagashima G, Itokawa H, Matsunaga A (2006). Astrocytes co-express aquaporin-1, -4, and vascular endothelial growth factor in brain edema tissue associated with brain contusion. Acta Neurochir Suppl.

[99] Plog BA, Nedergaard M. The glymphatic system in central nervous system health and disease: past, present, and future. 2018;13:379–94. 101146/annurev-pathol-051217-111018. 10.1146/annurev-pathol-051217-111018 PMC5803388 29195051

[100] Denver P, D’adamo H, Hu S, Zuo X, Zhu C, Okuma C (2019). A novel model of mixed vascular dementia incorporating hypertension in a rat model of Alzheimer’s disease. Front Physiol.

[101] Sweeney MD, Sagare AP, Zlokovic BV (2018). Blood–brain barrier breakdown in Alzheimer’s disease and other neurodegenerative disorders. Nat Rev Neurol.

[102] Luque FA, Jaffe SL (2007). Cerebrospinal fluid analysis in multiple sclerosis. Int Rev Neurobiol.

[103] Palomares JA, Tummala S, Wang DJJ, Park B, Woo MA, Kang DW (2015). Water exchange across the blood-brain barrier in obstructive sleep apnea: an MRI Diffusion-weighted pseudo-continuous arterial spin labeling study. J Neuroimaging.

[104] Huang J, Li J, Feng C, Huang X, Wong L, Liu X (2018). Blood-brain barrier damage as the starting point of leukoaraiosis caused by cerebral chronic hypoperfusion and its involved mechanisms: effect of agrin and aquaporin-4. Biomed Res Int.

[105] Hase Y, Chen A, Bates LL, Craggs LJL, Yamamoto Y, Gemmell E (2018). Severe white matter astrocytopathy in CADASIL. Brain Pathol.

[106] Lin Z, Li Y, Su P, Mao D, Wei Z, Pillai JJ (2018). Non-contrast MR imaging of blood-brain-barrier permeability to water. Magn Reson Med.

[107] Wengler K, Bangiyev L, Canli T, Duong TQ, Schweitzer ME, He X (2019). 3D MRI of whole-brain water permeability with intrinsic diffusivity encoding of arterial labeled spin (IDEALS). Neuroimage.

[108] Gregori J, Schuff N, Kern R, Günther M (2013). T2-based arterial spin labeling measurements of blood to tissue water transfer in human brain. J Magn Reson Imaging.

[109] Shao X, Zhao C, Shou Q, St Lawrence KS, Wang DJJ (2023). Quantification of blood–brain barrier water exchange and permeability with multidelay diffusion-weighted pseudo-continuous arterial spin labeling. Magn Reson Med.

